# O-44 - UNDERSTANDING SEXUALLY TRANSMITTED SHIGELLA CLUSTERS TO HELP CONTROL SPREAD

**DOI:** 10.1093/pubmed/fdag046.039

**Published:** 2026-07-09

**Authors:** Ms Holly Fountain, Ian Simms, Hannah Charles, Katy Sinka

**Affiliations:** Blood Safety, Hepatitis, STIs and HIV Division, UK Health Security Agency; Blood Safety, Hepatitis, STIs and HIV Division, UK Health Security Agency; Gastrointestinal Infections, Food Safety & One Health Division, UK Health Security Agency; Blood Safety, Hepatitis, STIs and HIV Division, UK Health Security Agency

## Abstract

**Background:**

Since the emergence of sexually transmitted Shigella among gay, bisexual and other men who have sex with men (GBMSM) circa 2008, cases have been increasing, often within persistent genomic clusters.

**Objectives:**

Describe sexually transmitted Shigella clusters to better understand transmission and help control spread.

**Methods:**

Data on Shigella diagnoses (2016-2025) was extracted from the Gastro Data Warehouse (GDW) and enhanced by questionnaire data (ESQ) from health protection teams. GDW data (available for 44% of all reports) provides genomic data for cluster typing and antimicrobial resistance (AMR). Adult (>15 years) males without reported travel were used as a proxy for sexual transmission (presumptive GBMSM [pGBMSM]). Clusters were defined as ≥10 isolates with the same 10 SNP address. GBMSM-associated clusters contained at least 85% diagnoses among pGBMSM.

**Results:**

GDW reported 6,016 Shigella diagnoses among pGBMSM in the last decade, increasing from 382 (2016) to 968 (2025). 57% (3,455/6,016) were in GBMSM-associated clusters. These were a mix of S. flexneri and S. sonnei, with S. flexneri most prominent (2,228/3,455 diagnoses, 19/27 clusters). 97% showed AMR markers.

Eight GBMSM clusters have persisted for an average (range) of 6.25 years (3-9 years) with average (range) 348 diagnoses (140-767), accounting for 80% of diagnoses in GBMSM-associated clusters (2,780/3,455). These clusters comprised: 87% pGBMSM (no documented travel), 3% ESQ-documented GBMSM who had travelled, 5% adult men (no documented sexual orientation) who had travelled, 3% women and 1% children. Of GBMSM travellers, 78% travelled to Europe (47% to Spain).

**Conclusions:**

GBMSM-associated clusters persist, remaining predominantly within this sexual network, which has an international reach. Ongoing transmission of strains with high levels of AMR is concerning, and, pending availability of a vaccine (several are in development), must rely on addressing low levels of awareness and advice on self-care and hygiene to reduce and prevent transmission.

